# A novel T-cell proliferation-associated regulator signature pre-operatively predicted the prognostic of bladder cancer

**DOI:** 10.3389/fimmu.2022.970949

**Published:** 2022-09-23

**Authors:** Jian Hou, Xiangyang Wen, Zhenquan Lu, Guoqing Wu, Guang Yang, Cheng Tang, Genyi Qu, Yong Xu

**Affiliations:** ^1^ Department of Urology, Zhuzhou Central Hospital, Zhuzhou, China; ^2^ Division of Urology, Department of Surgery, The University of Hongkong-Shenzhen Hosipital, Shenzhen, China

**Keywords:** bladder cancer, single cell sequencing, T-cell proliferation regulator, predictive gene signature, prognostic

## Abstract

**Background:**

Bladder cancer (BCa) is a remarkably malignant and heterogeneous neoplastic disease, and its prognosis prediction is still challenging. Even with the mounting researches on the mechanisms of tumor immunotherapy, the prognostic value of T-cell proliferation regulators in bladder cancer remains elusive.

**Methods:**

Herein, we collected mRNA expression profiles and relevant clinical information of bladder cancer sufferers from a publicly available data base. Then, the LASSO Cox regression model was utilized to establish a multi-gene signature for the TCGA cohort to predict the prognosis and staging of bladder cancer. Eventually, the predictive power of the model was validated by randomized grouping.

**Results:**

The outcomes revealed that most genes related to T-cell proliferation in the TCGA cohort exhibited different expressions between BCa cells and neighboring healthy tissues. Univariable Cox regressive analyses showed that four DEGs were related to OS in bladder cancer patients (p<0.05). We constructed a histogram containing four clinical characteristics and separated sufferers into high- and low-risk groups. High-risk sufferers had remarkably lower OS compared with low-risk sufferers (P<0.001). Eventually, the predictive power of the signature was verified by ROC curve analyses, and similar results were obtained in the validation cohort. Functional analyses were also completed, which showed the enrichment of immune-related pathways and different immune status in the two groups. Moreover, by single-cell sequencing, our team verified that CXCL12, a T-lymphocyte proliferation regulator, influenced bladder oncogenesis and progression by depleting T-lymphocyte proliferation in the tumor microenvironment, thus promoting tumor immune evasion.

**Conclusion:**

This study establishes a novel T cell proliferation-associated regulator signature which can be used for the prognostic prediction of bladder cancer. The outcomes herein facilitate the studies on T-cell proliferation and its immune micro-environment to ameliorate prognoses and immunotherapeutic responses.

## Introduction

Bladder cancer (BCa) is the 9th most commonly seen tumor across the globe and ranks 13th in terms of tumor mortality, and 10-15% of patients encounter metastasis at the time of diagnosis (1). Although surgical treatment and postoperative Bacillus Calmette-Guerin (BCG) infusion and other immunotherapies have been used in the clinical management of bladder cancer (2), bladder cancer still exhibits high recurrence and metastasis rates due to its remarkable heterogeneity and genomic instability (3, 4). About 50%-70% of NMIBC patients will experience recurrence within 5 years, with 10%-30% of patients progressing to an aggressive form (5, 6). Immune-checkpoint inhibitors (ICIs) that target the programmed death-1 (PD-1)/programmed death ligand 1 (PD-L1) axis and cytotoxic T lymphocyte-associated antigen-4 (CTLA-4) are emerging as valid salvage treatments for sufferers displaying chemotherapy resistance, but the response rate to therapy is relatively low (21%) (7). Therefore, it’s imperative to search for new therapeutic targets or biomarkers regarding the efficacy of ICIs associated with BCa immunotherapy. Cancer-infiltrating immunocytes, like modulatory T cells (Tregs), macrophagus, mast cells, and B cells, may influence the equilibrium between anti-cancer immunity and immuno-evasion in MIBC (8–11). Studies on BCG-refractory bladder cancer suggest that BCG tolerance might be mediated by an intricate causal link of immune-evasion (12). Several studies have identified that tumors may evade antitumor immunity through immune-checkpoint paths regulating T cell stimulation. Substantial immune-checkpoint molecules participate in the such causal link, like CTLA-4, PD-1 and its ligands PD-L1 and PD-L2 (13). Cancer immunotherapy targeting tumor-specific T cells can benefit tumor sufferers, whereas the clinical effectiveness changes remarkably in different tumor types. Cancer-infiltrating T cells frequently develop into a dysfunction status. T cell failure, and the anti-tumor function of effector T cells is modulated by several factors, like modulatory T cells (Treg). The changes of status and abundance of T cells depend on the tumor microenvironment (TME) of diverse tumor types, which might influence clinical results, like drug responses to immunotherapies (14). The specific mechanisms by which different subtypes of T lymphocytes in the tumor microenvironment modulate each other and their roles in the prognosis of BCa immunotherapy remain elusive.

In this study, a T lymphocyte proliferation regulatory factor-associated prognostic model was systematically evaluated to reveal the correlation with prognostic and clinicopathological characteristics of BCa patients. First, a column line graph containing T-lymphocyte proliferation regulatory factor characteristics and clinical factors was created to forecast survival in those sufferers. Then, our team verified the genes in the signature were expressed differently in normal bladder tissue and bladder tumor tissue by immunohistochemistry (IHC). Eventually, we verified the communication crosstalk between cells in the bladder tumor microenvironment by single cell sequencing. All in all, this research offers enlightenment regarding the modulatory causal links through which T lymphocytes promote BCa and might ameliorate the validity of personalized therapy and prognostic assessment.

## Materials and methods

### Datasets and pre-processing

The RNA-seq profiles of BCa (n=414) and normal bladder specimens (n=19) from the TCGA data portal (https://tcga-data.nci.nih.gov/tcga/) (Level 3 data, FPKM value) were acquired. The data type was set to “Gene Expression Quantification “ and the work flow type was “HTSeq-FPKM”. For further analysis, our team first normalised expression profiles to transcripts per kilobase million values, and each analysis was completed *via* the R software (4.1.1). Gencode (version 26) GTF files were obtained through Ensembl (http://asia.ensembl.org) for annotation and differentiation of mRNAs and lncRNAs [25], while sex, age, clinical staging, survival rates were acquired from the TCGA data portal, after the removal of specimens with no clinical data or with a survival duration of 0 day. Overall, 408 BCa samples and 19 normal bladder specimens were selected. The full clinical characteristics of BCa sufferers are shown in **Supplementary Table 1**. Internal standardisation was completed through the “limma” package. Afterwards, difference analyses were completed through the “Deseq2” R package. Then, 35 T cell proliferation regulatory factors were retrieved from a previous literature (15). This is the first article defining the definition of T-cell proliferation regulators (TCRs). The 35 genes obtained were used in our subsequent analysis, and they are displayed in **Supplementary Table 2**.

### Functional and pathway enrichment analyses

DEG’s putative biological processes, cellular components, and molecular activities were investigated using GO enrichment and KEGG pathway analysis. The signal pathways were strongly correlated with what David (https://DAVID.ncifcrf.gov/) found. This research makes use of annotation, visualization, and a large discovery database (David did a functional enrichment analysis on TCRs variables. Furthermore, using the R package “cluster Profiler” and data from the Kyoto Encyclopedia of Genes and Genomes (KEGG), functional analysis of biological processes (BP), molecular functions (MF), and cellular components (CC) regulated by macrophage phagocytosis regulators was done. The cut-off for P values was established at P< 0.05.

### Development of a T cell proliferation related prognostic risk model

TCGA-BLCA sufferers were stochastically separated into learning and verification groups *via* a stochastic grouping approach. Then, univariable Cox analyses of OS were completed to identify TCR-associated genes with prognosis significance. An interplay net of overlapped prognosis TCRs-associated genes was produced through the STRING data base 11.0 (16). The univariate Cox was applied to investigate the prognostic value of DEFAGs. LASSO Cox regression analysis was then applied to minimize the risk of overfitting, contributing to variable selection and regularization (17, 18). Eventually, we established a prognostic model by employing the multiple stepwise Cox regression. The algorithm of each BLCA patient was constructed as follows:Riskscore = esum (the expressing level of every gene×relevant coefficient). Sufferers were separated into the risk_high_ and risk_low_ groups as per the mid-value of the risk score. PCA was completed using the “prcomp” function of the “stats” R package as per the genetic expressions in the hallmark. For the survival analysis of each gene, the optimal cutoff expression value was determined by the “surv_cutpoint” function of the “survminer” R package. ROC curve analysis over time was completed using the “survROC” R package to assess the prediction power of the gene hallmark.

### Nomogram construction and validation

Univariable and continuous multivariable Cox regressive analysis was performed to identify whether the T cell proliferation regulator (TCR)-associated model was independent of certain clinic features through survival R packages. Afterwards, a nomogram was established on the basis of the multivariable Cox regressive coefficients of TCR features in the TCGA learning cohort and it was created according to clinical variables. The consistency index (C-index) was computed to verify the prediction ability of the column line plots, and correction curves were drawn to check the consistency between the forecasted 1-, 3-, and 5-year OS possibilities and the real observational results (as per bootstrap 1000 iterative resampling validation).

### TME cell infiltration

Our team utilized the CIBERSORTx arithmetic and EPIC to realize the quantification of immunocytes. For CIBERSORTx, the normalised genetic expression information was uploaded to the online platform *via* LM22 signature and 1,000 permutations (19). EPIC is an online platform for studying mass cytometry data from immunocytes in a standardised way (20). Cancer purity scoring was speculated *via* the “ESTIMATE” package (21, 22).

### Mutation SNP and copy number variation analyses

To explore the diversity in SNP expression between riskhigh and risklow patients, our team acquired and studied the SNP data of BLCA *via* the maftools package (23), and the Top 20 genetic variants were visualised *via* waterfall plots with the 10 core genes described in Section 2.12. Tumor mutation burden (TMB) was the sum of somatic mutations per megabase in each cancer specimen. Hence, our team computed the quantity of genetic variants in each cancer specimen to identify the TMB. Afterwards, our team performed statistic assay between groups to identify the TMB in riskhigh and risklow patients.

CNV segment files were acquired and analyzed to acquire the biomarker files and the files were uploaded to the Gene Pattern Gistic 2.0 module for CNV analyses. The data base default was chosen for analyzing variables, and the maftools package (23) was utilized to visualise the outcomes of CNV analyses.

### Drug sensitivity analysis

Chemotherapy susceptibility and immune therapy reaction forecast. To forecast susceptibility between high and low TCR risk groups, our team utilized the pRRophetic R package (0.5) to acquire IC50 values *via* establishing a ridge regressive model with 10-fold cross-verification (24, 25). Multiple commonly seen anti-tumor medicines (cisplatin, docetaxel, gefitinib, gemcitabine, pazopanib, sunitinib) and their gene profiles were acquired from the biggest public pharmacogenomic data base: the Genomics of Drug Sensitivity in Cancer (GDSC) (https://www.cancerrxgene.org/) (26). Moreover, the Tumor Immune Dysfunction and Exclusion (TIDE) (http://tide.dfci.harvard.edu/) arithmetic was utilized to forecast the reaction to immune-checkpoint blockage treatment between these 2 groups (27).

### Single-cell analysis of the origin of gene expression in the signature

To reveal if the expression levels of genes in the hallmark are associated with T cells in the TME, the TISCH data base (http://tisch.comp-genomics.org/home/ ) was utilized (28). TISCH is a TME-focused scRNA sequencing data base providing cell type annotation details at the single-cell level, which allows us to investigate TME in different cancer types including BCa.

### Human protein atlas database and IHC validation

The protein expression levels of those TCRs-related hallmark genes in BCa samples was studied in the HPA on-line data base (https://www.proteinatlas.org/) intending to produce a HPA *via* integratedomics techniques (29).

## Result

### Overview of gene variations and expression variants of TCRs-associated genes in BCa

**Figure 1:**
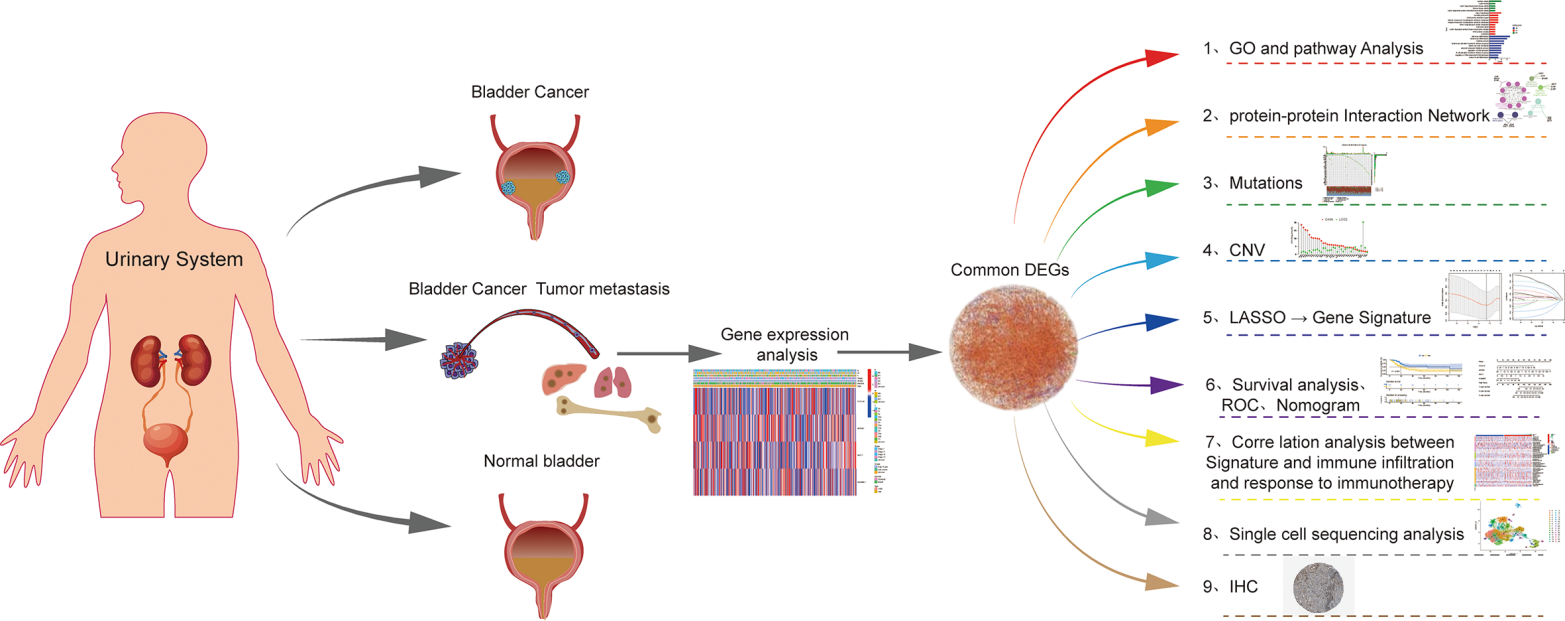
The flowchart for this article.

**Figure 2:**
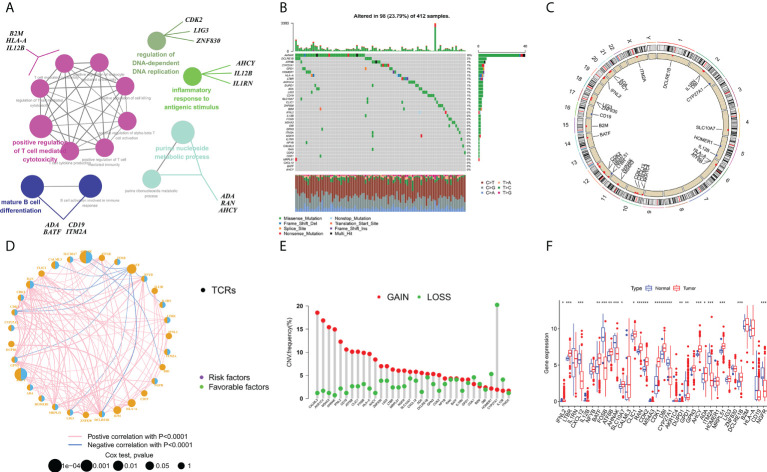
Characterization and differences of T cell proliferation regulator-associated regulators in BCa. **(A)** A collection of potential biological interactions with TCR-associated modulators from the STRING platform. **(B)** Mutation mapping of 412 BCa sufferers from the TCGA-BLAC cohort. Each waterfall plot represents data about every TCR-associated mutation. Relevant colors are annotated at the bottom, which means different mutation types. The bar line graph shows the mutation burden. The correct number indicates the mutation frequency respectively. **(C)** CNV change position of TCRs on chromosome TCGA cohort. CNV, copy number change. **(D)** Interactions among TCRs in BCa. The circle size denotes the effect of every modulator on prognosis, and contrasts were completed *via* the log-rank test (p < 0.05, p < 0.001, p < 1E-05 and p < 1E-08), and blue connecting lines represent negative correlations. **(E)** TCGA cohort associated with TCRs in CNV frequency. The height of the bars shows the proportion of different types of CNV. **(F)** Expression of TCRs between normal and bladder cancer tissues (Wilcox test, ^∗^P < 0.05;^∗∗^P < 0.01;^∗∗∗^P < 0.001;P < 0.0001;ns, not statistically significant).

The flow chart for this article is shown in **Figure 1**. The STRING platform was utilized to analyze the potential biofunctional network associated with 35 regulatory factors related to T-cell proliferation (**Figure 2A**). We focused on regulatory factors related to immune response and T-cell proliferation. At the gene level, 98 out of 412 specimens (56.79%) displayed variants in regulatory factors associated with T cell proliferation, among which AHNAK showed the highest mutation frequency (**Figure 2B**). Our team also identified CNVs in 34 regulatory factors associated with T cell proliferation, and we identified 34 alterations in regulatory factors correlated with CNVs on chromosomes (**Figures 2C, E**). **Figure 2D** demonstrates the regulatory relationship between prognosis-related TCRs and risk factors for bladder cancer. Compared to normal samples, where the expression levels of IFNL2, CXCL12, IL12B, FOSB, AHNAK, CYP27A1, GPD1, ITM2A, CD19 and NGFR were decreased, the expression levels of all other genes were elevated. Interestingly, our study also identified a reciprocal regulatory relationship between TCRs associated with bladder cancer prognosis (**Figure 2F**). The analytical results in the present research unveiled that the expression levels of T-cell proliferation-associated regulatory factors were associated with BCa, which hence unveiled that they might show the diverse characteristics of sufferers. Afterwards, we found that the majority of TCRs were related to worse survival in bladder cancer according to the results of survival analysis (**Supplementary Figure 1**).

### Functional enrichment analysis of T cell proliferation regulator-associated regulators in BCa

**Figure 3:**
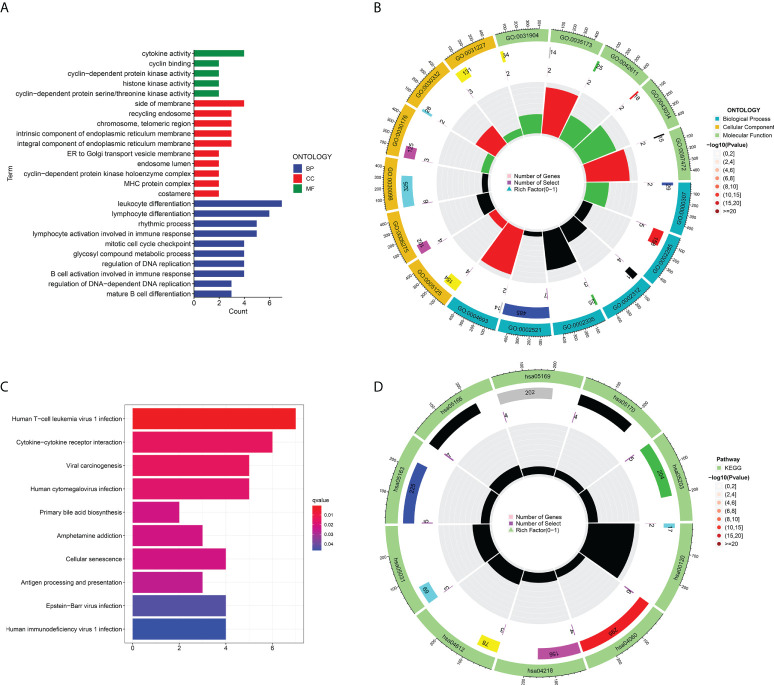
Functional analysis of T cell proliferation-associated regulatory factors: **(A, B)** the enrichment analysis of T cell proliferation-associated regulatory factors (BP, CC, MF). **(C, D)** the KEGG pathway enrichment analysis.

To analyze the potential roles of DEGs, GO analyses were performed through the DAVID website and we visualised the data in R. Regarding biological processes (BPs), the results revealed that genes related to TCRs were mainly enriched in cytokine activity, cyclin binding, cyclin-reliant protein kinase activity, histone kinase activity and cyclin-reliant protein serine/threonine kinase activity. Regarding cell composition (CCs), they were mainly enriched in side of membrane, recycling endosome, chromosome, telomeric region, inherent constituent of endoplasm reticulum membrane and MHC protein complex. In addition, in terms of molecular function (MF), TCRs were mainly enriched in leukocyte differentiation, lymphocyte differentiation, rhythmic process, mitochondrial cell cycle checkpoint and B cell activation involved in immune response. (**Figures 3A, B**). Then, we further analyzed the KEGG pathway enriched in TCR-related genes using the DAVID online tool and visualised it by R language. We found that TCRs were mainly enriched in human T-cell leukemia virus 1 infection, cytokine-cytokine receptor interaction, viral carcinogenesis, human cytomegalovirus infection, antigen processing and presentation, and HIV 1 infection signaling pathways (**Figures 3C, D**).

### Establishment of a prognosis model in TCGA cohort

**Figure 4:**
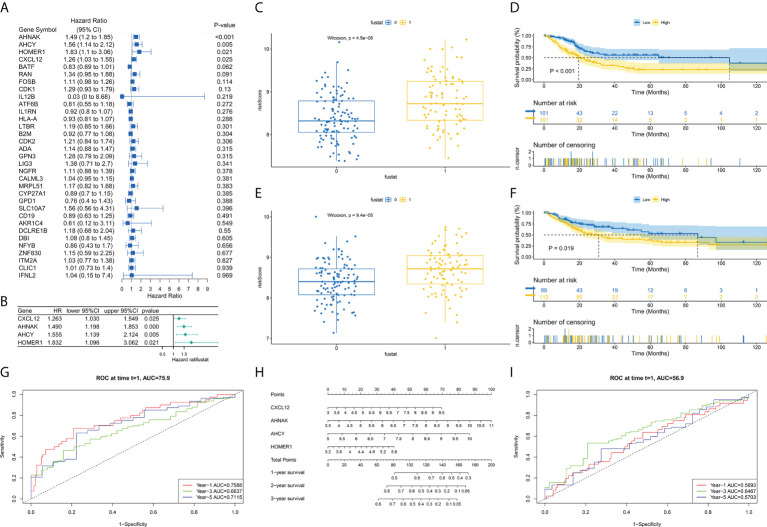
Development of TCRs-related prognostic signature. **(A)** Univariable Cox hazard analyses were completed on TCRs pairs. **(B)** LASSO regression of TCRs for establishing prognosis. **(C, E)** Difference in survival status between risk_high_ and risk_low_ sufferers in learning and verification groups. **(D, F)** Survival analysis shows that survival outcomes are significantly different between risk_high_ and risk_low_ sufferers in learning and verification groups. **(G, I)** ROC curves at 1, 3 and 5 years are described for learning and verification groups. **(H)** Predictive efficacy of nomogram validation model.

First, we randomly divided TCGA-BLCA patients into the training and validation groups. To build a prognostic model, the expression profiles of the 35 aforesaid genes were studied *via* LASSO Cox regression analyses. We obtained a risk model constructed from four TCRs associated with bladder cancer prognosis (**Figures 4A, B**). In survival analyses, high expression level of each gene was found to be related to poor prognosis based on the optimum cutoff value (modified P*0.05, **Figures 4C, E**). Risk scores were computed through: Risk score = (0.279*the expression level of CXCL12 + 0.351*the expression level of AHNAK + 0.455*the expression level of AHCY + 0.440*the expression level of HOMER1). Sufferers were separated into risk_high_ (n=213) or risk_low_ groups (n=190) as per median cutoff values. In the TCGA-BLCA training cohort, we found that patients in the high-risk score group possessed poorer survival rates. Consistently, Kaplan-Meier (K-M) curves showed that risk_high_ sufferers displayed remarkably shorter OS in contrast to risk_low_ sufferers (**Figures 4D, F**. P<0.001). Surprisingly, in the TCGA-BLCA validation cohort, we observed the same results. The prediction ability of the risk scoring for OS, assessed by the ROC curve, reached an AUC of 0.759 at 1 year, 0.664 at 3 years and 0.712 at 5 years in the training group over time (**Figures 4G, I**). In the validation group, we also obtained a better prognostic efficacy. Its AUC attained 0.5693 at 1 year, 0.6467 at 3 years, and 0.5703 at 5 years, respectively. Surprisingly, considering the significance of risk scoring in forecasting the prognoses of BCa sufferers, our team afterwards investigated its clinical potential. Our team established a nomogram having four clinical characteristics that are readily available and normally considered to exert an influence on the prognoses of BCa, and such nomogram had the ability to predict the 1-, 2-, and 3-year OS for BCa sufferers (**Figure 4H**). Collectively, the nomograph has satisfactory forecast ability. We found that four genes showed differential expression in samples from different risk groups (**Figure 6A**).

### Differences in TCR-related signature and TME infiltration and analysis of immunotherapy correlation

Tumor-associated fibroblasts, extracellular matrix, immune cells, various growth factors, inflammatory factors (characterized by special physicochemical characteristics), cancer cells, etc. exist in the tumor microenvironment. The microenvironment significantly affects the diagnosis of tumors, survival outcomes, and response degree toward clinical treatment. The outcomes revealed that risk score was tightly associated with immunocyte infiltration. To unveil the association between the risk scoring and immunity status, our team quantified the enrichment scoring of different immunocyte sub-populations, correlated biofunctions or pathways *via* ssGSEA. Interestingly, the outcomes revealed that CXCL12 and AHNAK were related to most immunocyte infiltration in a positive manner, while AHCY and HOMER1 were related to most immunocyte infiltration in a negative manner (**Supplementary Figure 2**). Further analysis of the results similarly confirmed that risk scoring was related to most immunocyte infiltration. **Figure 6A** demonstrated the association between the expressions of the four TCRs in each sample and the clinic features of patients.

### Correlation of risk score with tumor mutations and gene regulation

**Figure 5:**
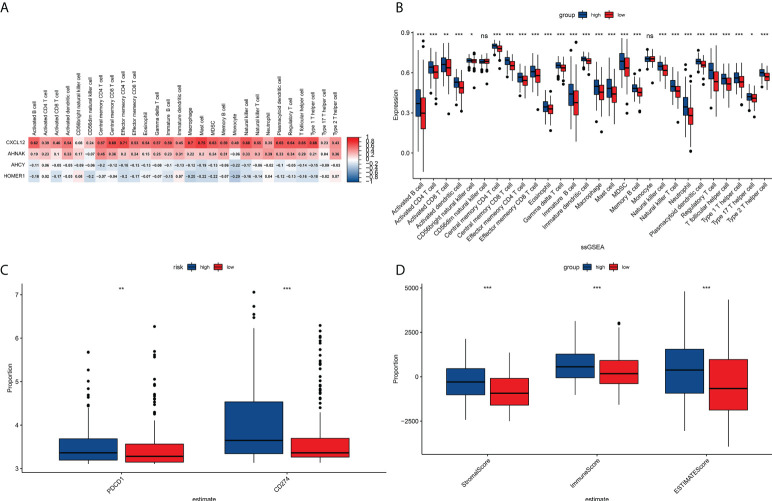
TME immunocyte infiltration features and immune constituents among different risk groups. **(A)** Heat map showing the correlation analysis between four prognostically relevant TCRs and 28 immune cell subpopulation infiltrates. **(B)** Relative richness of every infiltration cell type expressed between risk_high_ and risk_low_ sufferers. **(C)** Correlation analysis of signature with PD1/PDL1 treatment. **(D)** Differences between stroma scoring, immunity scoring and Estimate scoring between risk_high_ and risk_low_ sufferers. *P<0.05, **P<0.01, ***P<0.001, ns: P≥0.05.

**Figure 6:**
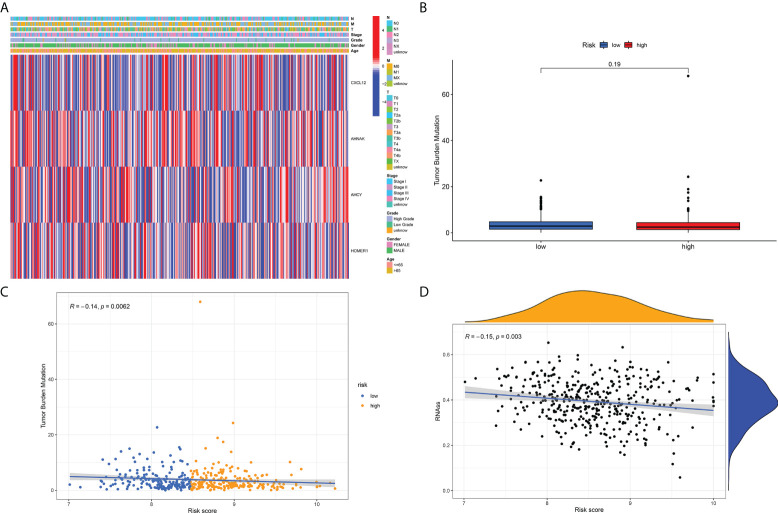
**(A)** Distribution of the risk score, the associated survival data and the mRNA expression heat map in the TCGA dataset. **(B)** Analysis of differences in TMB scores between high and low risk score groups. **(C)** Risk score and TMB correlation analysis. **(D)** Correlation analysis of risk score and tumor stem cell index.

As per those outcomes, our team discovered that risk scores were vital for clinical forecast. Subsequently, we explored if risk scores could facilitate clinical therapy, particularly immune therapy. The infiltration of TME cells with diverse risk score was studied (**Figure 5B**). The outcomes revealed that risk score was tightly associated with immunocyte infiltration. To unveil the association between risk scoring and immunity status, our team quantified the enrichment scoring of diverse immunocyte sub-populations, associated biofunctions or paths *via* ssGSEA. Interestingly, the our revealed that CXCL12 and AHNAK were related to the majority of immunocyte infiltration in a positive manner, whereas AHCY and HOMER1 were related to the majority of immunocyte infiltration in a negative manner (**Figure 5A**). Further analysis of the results similarly confirmed that risk score was related to the majority of immunocyte infiltration. The immunity scores from ESTIMATE analysis decreased with the increasing risk scoring, while stroma scoring displayed contradictory effects. Those data suggested that risk_low_ sufferers had stronger immune responses than risk_high_ sufferers. Diversities in TME cells might be the primary cause for the inhomogeneity of risk scoring. Cancers attracting more T-cell infiltration are referred to as “hot cancers” and are more susceptible to immune therapy, which hence leads to superior immunotherapeutic results (30). Recent studies have confirmed that sufferers with greater somatic TMB display reinforced responses, longterm survival, and persistent clinical benefits after immune-checkpoint blockage (31). Fortunately, we explored TMB across risk scoring and found a strong correlation between risk score and TMB (**Figures 6B, C**). These results suggest that risk score may forecast the prognoses of BCa sufferers and may show the reaction to immune therapy.

Previous studies have suggested that T-cell proliferation-related regulators might reflect the effects of immune therapy. There are proofs supporting the fact that sufferers with high TMB status have a durable clinical response to anti-PD1/PD-L1 immune therapy (31). Surprisingly, we first examined changes in the expression of immune-checkpoints. Our team contrasted the diversities in the expression levels of immune checkpoint genes in the TCGA-BLCA cohort (**Figure 5C**). Next, we analyzed the roles of risk scoring in immunotherapy response, and the results showed that the cohort with higher scores had better immunotherapy outcomes (**Figure 5D**). When the results of risk scoring and stem cell index were analyzed, a positive association was discovered between them (**Figure 6D**). Those outcomes explain the latent role of risk scoring in immune therapy and provide some evidence that risk scoring can be utilized as a prediction factor of immune therapy response.

### Correlation analysis of risk score and drug sensitivity analysis

To further explore the effect of TCR-related prognostic models on the response to bladder cancer drug therapy, we performed a correlation analysis by analyzing risk score and bladder treatment drugs. Surprisingly, the outcomes revealed that risk score was remarkably correlated with drug treatments as displayed in **Supplementary Figure 3**. Those outcomes suggest that TCR-related genes might be related to BCa drug resistance and might be latent treatment targets for Bladder cancer drug therapy.

### Single-cell RNA sequencing analysis, clustering and marker identification

**Figure 7:**
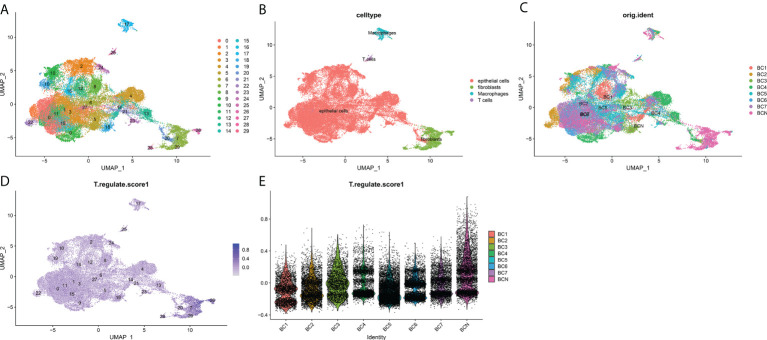
Overview of single cells from BCa and non-malignant tissues. **(A)** 29 major cell types, **(B)** T cells, epitheliums, fibroblasts, and macrophages are identified. **(C)** Identification of the distribution of various cells in tissues. **(D)** The deeper the color, the smaller the P value. **(E)** T cell proliferation regulatory factors among the expression of seven cell types.

**Figure 8:**
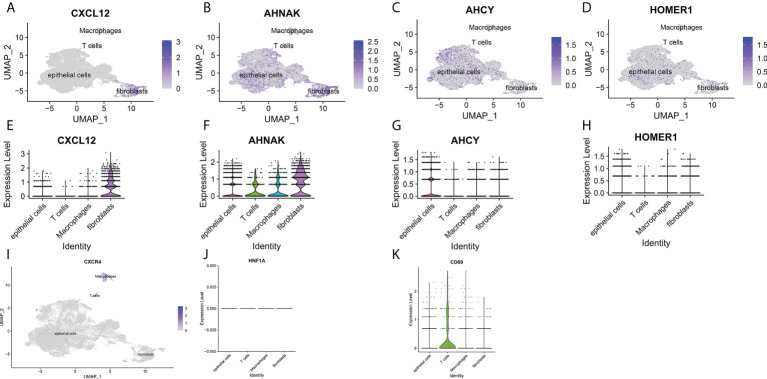
**(A-D)** UMAP plots of single cells for four prognosis-related T cell proliferation regulation factors. **(E-H)** Expression profiles of four prognosis-related T cell proliferation regulation factors in T cells, epithelial cells, fibroblasts and macrophages. **(I)** UMAP plots of single cells for specific receptors of CXCL12. **(J)** Expression profiles of T cell proliferation regulatory factor CXCL12 in regulatory T lymphocytes. **(K)** Expression profile of T cell proliferation regulatory factor CXCL12 in depleted T lymphocytes.

To further explore the mechanism of the effects of T-cell proliferation factors on the tumor microenvironment of bladder cancer. We showed 29 cell back markers by single cell sequencing analysis (**Figure 7A**). Their cell types were mainly fibroblasts, T cells, macrophages and epithelial cells (**Figure 7B**). Surprisingly, T-cell proliferation factor was specifically expressed in paracancerous tissues, which were mainly fibroblasts (**Figure 7C**). Likewise, further analysis showed that BCN had the highest score, that is, the highest score of paraneoplastic tissues (**Figures 7D, E**). Interestingly, CXCL12 was mainly enriched in fibroblasts in paraneoplastic tissues (**Figures 8A-H**). Recent studies have confirmed that CXCL12 is a regulatory factor located in the cytoplasm, whereas the other three are expressed in the nucleus. Therefore, we focused on CXCL12. We conjectured that tumore-related fibroblasts in the BCa TME regulate T-cell proliferation in the TME by the paracrine secretion of CXCL12, thereby influencing tumorigenesis and progression. To test our conjecture, by further analysis, we found that CXCR4, a specific receptor for CXCL12, was expressed in T cells and macrophages in tumor tissues (**Figure 8I**). It is puzzling that T cell proliferation in tumor tissues promotes tumor progression. By testing Treg and T-cell failure markers (**Figure 8J**). Surprisingly, our results suggested that CD69 (a T cell failure-specific marker) was abnormally expressed in T cells in bladder tumor tissues, however, no significant expression was observed in other cells (**Figure 8K**). Taken together, we found that fibroblasts in bladder cancer parietal tissue promote bladder tumorigenesis and progression by the paracrine secretion of CXCL12 into the tumor microenvironment to bind specifically to CXCR4 receptors and promote the proliferative ability of depleted T cells in cancer tissues. This may be a latent new treatment target for bladder cancer immunotherapy.

### Expression features of TCR-associated signature genes at the protein level were investigated by HPA database and IHC analysis

**Figure 9:**
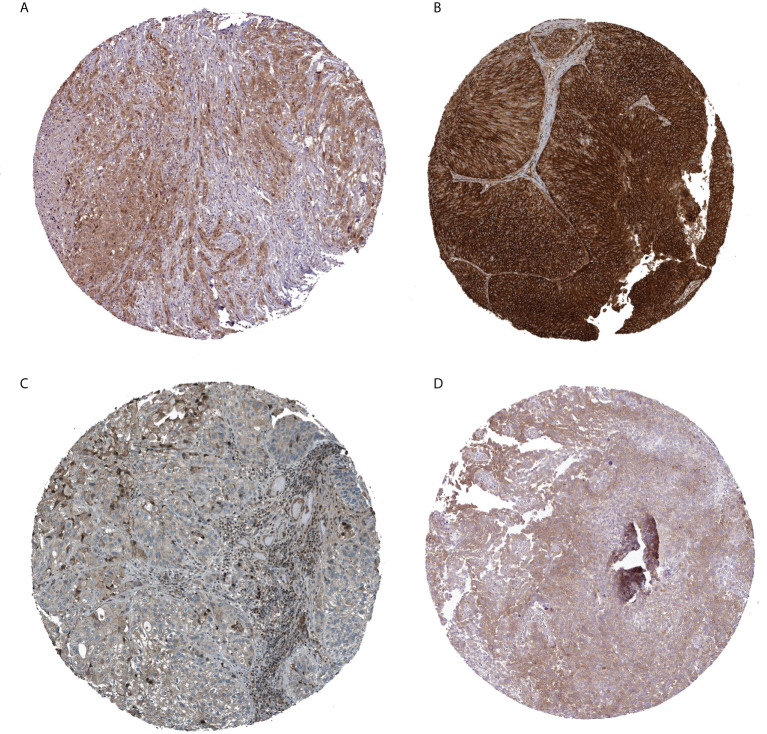
Immunohistochemical analysis of four prognostically relevant T cell proliferation-related regulatory factors (**A**: AHCY; **B**: AHNAK; **C**: CXCL12; **D**: HOMER1).

Finally, regarding the expression levels of BCa tissue proteins, immune histochemical outcomes from the HPA data base showed that the expression levels of CXCL12 and AHNAK were low in BCa tissue, whereas the protein expression of AHCH and HOMER1 was higher (**Figures 9A-D**).

## Discussion

BCa takes a heavy toll on Medical and Health Services worldwide, especially in Europe and North America (2). Most sufferers are confirmed with NMIBC at first diagnosis, where the tumor does not invade the muscular layer, and most cases can be treated by transurethral resection of bladder (TURB) or intravesicular BCG vaccine or other types of chemotherapy (5, 32, 33). However, a large proportion of patients with NMIBC will eventually develop into the MIBC stage (34), and the standard method for MIBC is radical cystectomy with or without neoadjuvant chemotherapy or radiotherapy. Even after treatment, approximately 50% of patients with MIBC will recur and die within 3 years (35). Recently, several researches have discovered that tumor cells can evade anti-tumor immunity by modulating T cell-activated immunity response mechanisms, and many immunity response related molecules participate in such causal link, like CTLA-4, PD-1 and its ligands PD-L1 and PD-L2 (13). Although the resulting cancer immunotherapies targeting cancer-specific T cells have brought substantial benefits to substantial tumor sufferers, the clinical effectiveness changes remarkably across tumor types. Therefore, a complete understanding of the mechanisms of T lymphocyte action in the immunotherapy of BCa is essential to guide the selection of immunotherapeutic regimens for BCa patients.

Herein, our team systematically explored the expression of T-cell proliferation regulators in BCa cancer tissue and their relationship with OS. Firstly, a new prognosis model consisting of four T-cell proliferation regulators in the TCGA-BCa cohort was constructed. Simultaneous function analyses showed that immunity-associated pathways were improved. Although it is currently well documented that T lymphocytes are vital for bladder oncogenesis and developmental process (36, 37), the exact mechanism of action remains unexplained. Surprisingly, the expression of T cell proliferation regulators was significantly different between cancer and neighboring non-tumor samples, and univariable Cox regression analyses revealed that a larger TCR was associated with OS in BCa patients. These results suggest a potential role of T lymphocyte proliferation regulators in BCa progression and the possibility of prognostic modeling with these T lymphocyte proliferation regulators.

The prognostic model proposed in this study consisted of four T cell proliferation regulators (CXCL12, AHNAK, AHCY and HOMER1).AHNAK is the largest protein in the human body and participates in cytoskeletal structure formation, muscle regeneration and calcium homeostasis (38). AHNAK is a cancer inhibitor protein and inhibits the developmental process of mammary and pulmonary cancer *via* enhancing the transforming growth factor-β (TGF-β) signal path (39, 40). The initiation of the T cell antigen acceptor in the course of immune antigen presentation requires the coordination of substantial signal transmission proteins and ionic pathways. AHNAK1 is a scaffolding protein and a key part of calcium signal transmission in the course of CD4 T cell stimulation (41), and AHNAK expression is tightly related to T cell responses. Several recent studies have verified the participation of AHNAK in the modulation of T-cell responses (42). Mazza et al. discovered that AHNAK-knockdown mice displayed damaged CD4+ T cell proliferative ability and decreased IL-2 generation upon *in vitro* activation *via* anti-CD3 antibodies (42). Nevertheless, the effects of AHNAK on immunomodulation and tumor immunotherapy are still elusive. Our results show that the expression of AHNAK is high in BCa nuclei and regulates oncogenesis and progression by modulating T cell responses in the bladder tumor microenvironment. Adenosine homocysteinase (AHCY) is a special enzyme and one of the most conservative proteins (43). Adenosine homocysteinase was originally defined as a cancer inhibitor (44). However, the function of AHCY as a cancer inhibitor appears to be cell type specific, as AHCY suppression is associated with antimigratory and anti-invasion activities in mammary carcinoma cells (45, 46) and related to enhanced apoptosis in highly aggressive neuroblastoma (47). In neuroblastoma, the expression level of AHCY was increased in MYCN-magnified cancer specimens and neuroblastoma lineage cells (48). Interestingly, AHCY knockout or medicine-mediated suppression caused an elevation in programmed cell death, particularly in MYCN-magnified neuroblastoma cells. In our study, AHCY was shown to be highly expressed in bladder tumors and it regulated oncogenesis and progression by affecting T lymphocyte responses in the tumor microenvironment. Homer-1 is a synaptic scaffolding protein that regulates glutamatergic synapses and spine morphogenesis (49). However, the role of Homer-1 in cancer immunotherapy is unclear. Herein, our team discovered that the expression of Homer-1 was high in bladder tumors and negatively correlated with patient OS. Chemotactic factor ligand C-X-C motif chemokine ligand 12 (CXCL12) is extensively expressed in a variety of tissues (50–52). The chemotactic factor CXCL12 is identified at commonly seen sites of cancer metastases and in animal models, and it’s expressed in circulation oncocytes (53). CXCR4 activates tumor metastases and its ligand CXCL12 is substantially generated. The interplay between CXCL12 and CXCR4 induces the forming of metastasis cancers. Moreover, CXCL12 hyper-methylation was discovered in multiple tumors, like stomach carcinoma (54), mammary carcinoma (55), colon carcinoma (56), pulmonary carcinoma (57), and prostate cancer (58), which suggested a possible role of CXCL12 in carcinogenesis. In our study, CXCL12 was found to be widely expressed in the fibroblasts of paraneoplastic tissues of bladder tumors by single cell sequencing analysis and immunohistochemistry. Its specific binding to CXCR4 receptors in T cells in the TME *via* paracrine secretion promotes the proliferation of depleted T cells, thus promoting bladder oncogenesis and progression, which may be a new latent treatment target for BCa immunotherapy. This discovery provides a new therapeutic direction for clinicians.

The most important contribution of our study is the identification of the association between T cell proliferation regulators and the TIME. Evidently, the intricate interactions between oncocytes and the TME are not only pivotal for cancer developmental process, but have a remarkable impact on immunotherapy effectiveness and OS (14, 59). Herein, function enrichment analyses showed that most TCR genes were sponged in signal paths like human T-cell leukemia virus infection, cytokine-cytokine acceptor interplay, and viral carcinogenesis. This demonstrates that TCR genes are vital for immunoregulation. Meanwhile, our team also found that risk_high_ sufferers had a greater proportion of stimulated memory CD4 T cells, activated CD8 T cells and mast cells, etc., which confirmed the role of T cell proliferation regulators in the modulation of cancer immunocyte infiltration. As the outcomes herein associate the TCR-related signature with BCa, those T cell proliferation regulators may be targeted for combination therapy with immune-checkpoint suppressors. Combining immune-checkpoint blockage with immune therapies like CTLA-4, PD-1 and PD-L1 suppressors is a prospective method for the treatment of multiple malignant tumors, and a stimulated TIME is related to satisfactory results with immune-checkpoint suppressor treatment (60, 61). Intriguingly, the expression level of PD-L1 was high in risk_high_ sufferers, which revealed that risk_high_ sufferers might have more benefits from anti-PD-L1 immune therapy, but the expression of CTLA-4 was high in risk_low_ sufferers, which revealed that risk_low_ sufferers might have more benefits from anti-CTLA-4 immune therapy. These outcomes offer novel enlightenment regarding cancer immune therapy.

Comparatively higher immune suppressive micro-environment and lower TMB have been shown to lead to immune therapy failures (62–64). We found that TMB was related to risk scoring in a negative manner and that risk_high_ sufferers had high immune scores and PD1 expression. These findings link the TCR-related signature for BCa immunotherapy to risk_high_ sufferers having better immunotherapy outcomes. This may also provide a latent treatment target for the prognosis of BCa immunotherapy. Interestingly, by single cell sequencing, we found that the expression of TCR-related gene CXCL12 was high, especially in fibroblasts in the TME, and it promoted the proliferation of T lymphocytes in the TME as well. This discovery is contrary to the widely recognized anti-cancer role of T lymphocytes. By searching for specific literature, we found that recent studies have revealed that the phenotype and abundance of T lymphocytes may vary greatly in the TME of various tumors, with a higher proportion of depleted T lymphocytes in the TME of patients with liver and colon cancer than in the TME of patients with lung cancer. In contrast, no significant expression was observed in the TME of patients with multiple myeloma (65–68). This difference in T lymphocyte infiltration is due to the fact that tumor-associated T lymphocyte phenotypic status and infiltration are influenced by multiple aspects (69). To identify the key factors effecting T lymphocyte status and proliferation in bladder cancer TME, we annotated single-cell data. From our results, we can see that CXCL12, which is highly expressed in the fibroblast cytoplasm, specifically binds to CXCR4, a specific receptor in T lymphocytes in TME, through paracrine action, promoting the proliferation of depleted T lymphocytes and thus evading immune surveillance to promote oncogenesis and progression. This finding might offer a novel treatment target for sufferers with advanced bladder tumors after they display immunotherapy resistance. It provides a new therapeutic direction for clinicians to follow.

All in all, this study identified 4 differently TCR-associated genes and successfully constructed an individualized signature, which proved to be significantly associated with prognoses of bladder cancer in both the derivation and validation datasets. The signature-based risk score can differentiate immunotherapy in high-risk sufferers exhibiting chemotherapy resistance. We also estimated the potential relationship among immunotherapy-related biomarkers, immune cell infiltration and immune-related pathways. In our future study, we may focus on the small molecule drugs related to TCR for BCa and try to verify TCR-associated gene as an oncogenic factor in BCa cell lines. This research was anticipated to provide new insights into ferroptosis for future work. Nevertheless, there are certain deficiencies in this research. Firstly, this paper is completed retrospectively on the foundation of publicly available databases, and the clinical effectiveness and steadiness of TCR-associated genetic signatures require more verification from larger prospective studies. Secondly, the biofunctions of genes have to be explained further by more experiments. Thirdly, the reaction of TCGA patients to immune therapy is based on algorithmic predictions, and the accurateness of the genetic hallmark has to be validated in actual immunotherapy cohorts.

## Conclusion

In conclusion, a T-cell proliferation regulator (TCR) associated signature was established to forecast the prognoses of bladder cancer pre-operatively. This signature which involves 4 TCR genes may have an association with oncogenesis, progression and metastasis in bladder cancer. This is one of the few studies that focus on the immunotherapeutic value of TCR associated with bladder cancer. Those outcomes offer a foundation for exploring the T cell proliferation regulator-related mechanism in BCa immunotherapy.

## Data availability statement

The datasets presented in this study can be found in online repositories. The names of the repository/repositories and accession number(s) can be found in the article/**Supplementary Material**.

## Author contributions

JH, XW, ZL and GW wrote the main manuscript text. GQ performed experiments. GY, CT and YX collected data. All authors contributed to the article and approved the submitted version.

## Acknowledgments

This work was supported in part by grants from Hunan Natural Science Foundation (#2021JJ41094).

## Conflict of interest

The authors declare that the research was conducted in the absence of any commercial or financial relationships that could be construed as a potential conflict of interest.

## Publisher’s note

All claims expressed in this article are solely those of the authors and do not necessarily represent those of their affiliated organizations, or those of the publisher, the editors and the reviewers. Any product that may be evaluated in this article, or claim that may be made by its manufacturer, is not guaranteed or endorsed by the publisher.

## Supplementary material

Supplementary Figure 1 Survival analyses of T cell proliferation-associated regulatory factors.

Supplementary Figure 2 Correlation analysis of signature with 28 immune cell infiltrates.

Supplementary Figure 3 Signature and drug sensitivity correlation analysis.

Supplementary Table 1 The clinical features of BLCA patients.

Supplementary Table 2 T-cell proliferation-associated regulaters.

## Abbreviations

TCRs, T-cell Proliferation-associated Regulators; BCa, Bladder cancer; OS, Overall Survival; NMIBC, non-muscle invasive bladder cancer; MIBC, muscle invasive bladder cancer; BCG, Bacillus Calmette-Guerin; BLCA, Bladder Urothelial Carcinoma; DEGs, Differential Expression Genes; FDR, false discovery rate; log2FC, log2 fold change; TCGA, The Cancer Genome Atlas; GO, gene ontology; BP, Biological Process; CC, Cellular Component; MF, Molecular Function; KEGG, Kyoto Encyclopedia of Genes and Genomics; GDSC, Genomics of drug sensitivity in cancer; CNV, Copy number variations; SNP, single nucleotide polymorphism; TMB, tumor mutational burden; IC50, half maximal (50%) inhibitory concentration; scRNA, Single Cell RNA-seq; HPA, the human protein atlas; ssGSEA, single sample gene set enrichment analysis; ICIs, Immune-checkpoint inhibitors; PD-1, programmed death-1; PD-L1, programmed death ligand 1; CTLA-4, cytotoxic T lymphocyte-associated antigen-4; CTL, cytotoxic T lymphocyte; Treg, Regulatory cells; TME, tumor microenvironment; IHC, immunohistochemistry.
