# Supplementary figures and images for: A novel T-cell proliferation-associated regulator signature pre-operatively predicted the prognostic of bladder cancer
Source: Front Immunol. 2022 Sep 23;13:970949. doi: 10.3389/fimmu.2022.970949 (PMC9539738; doi:10.3389/fimmu.2022.970949)

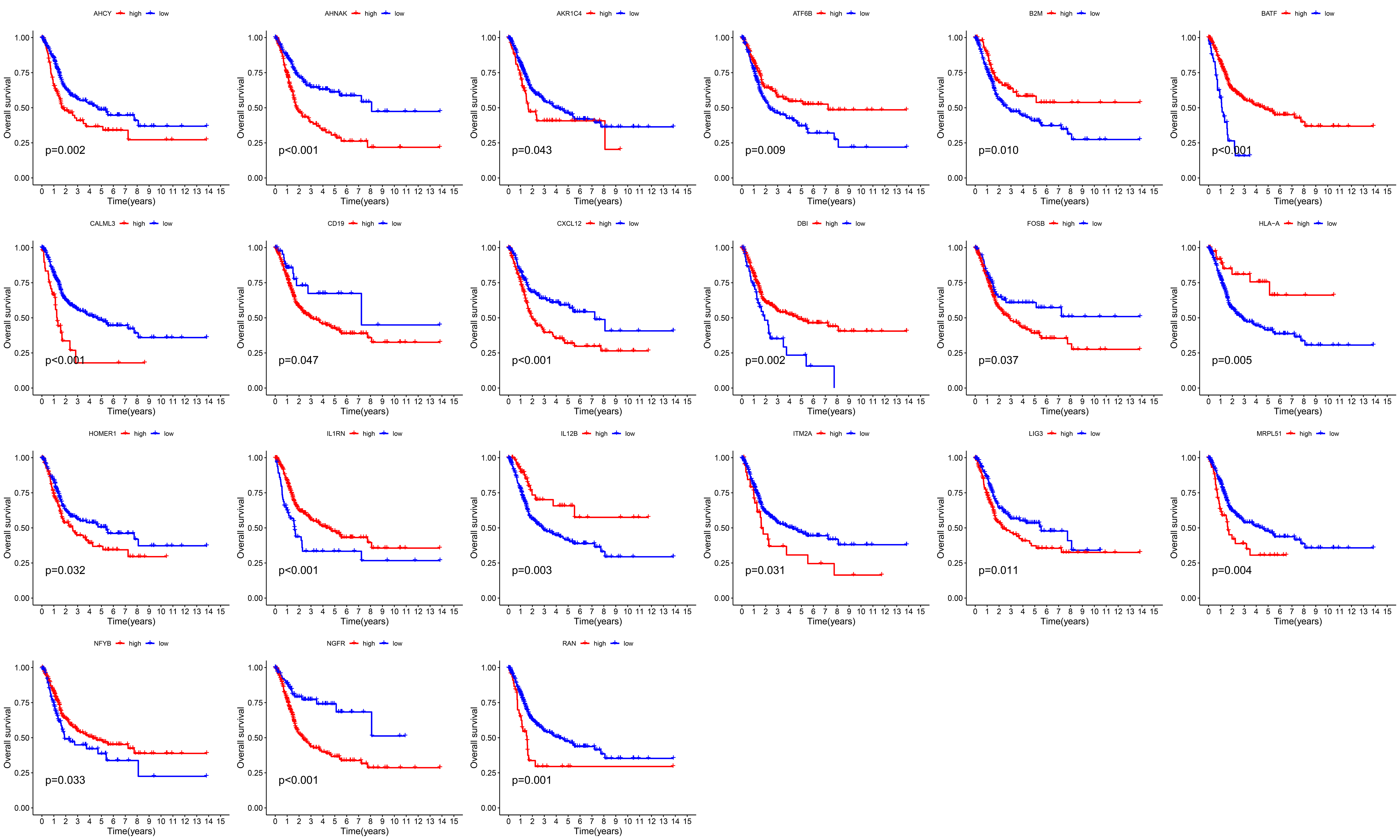

Supplement: Supplementary Figure 1 — Survival analyses of T cell proliferation-associated regulatory factors. [file Image_1.tif]

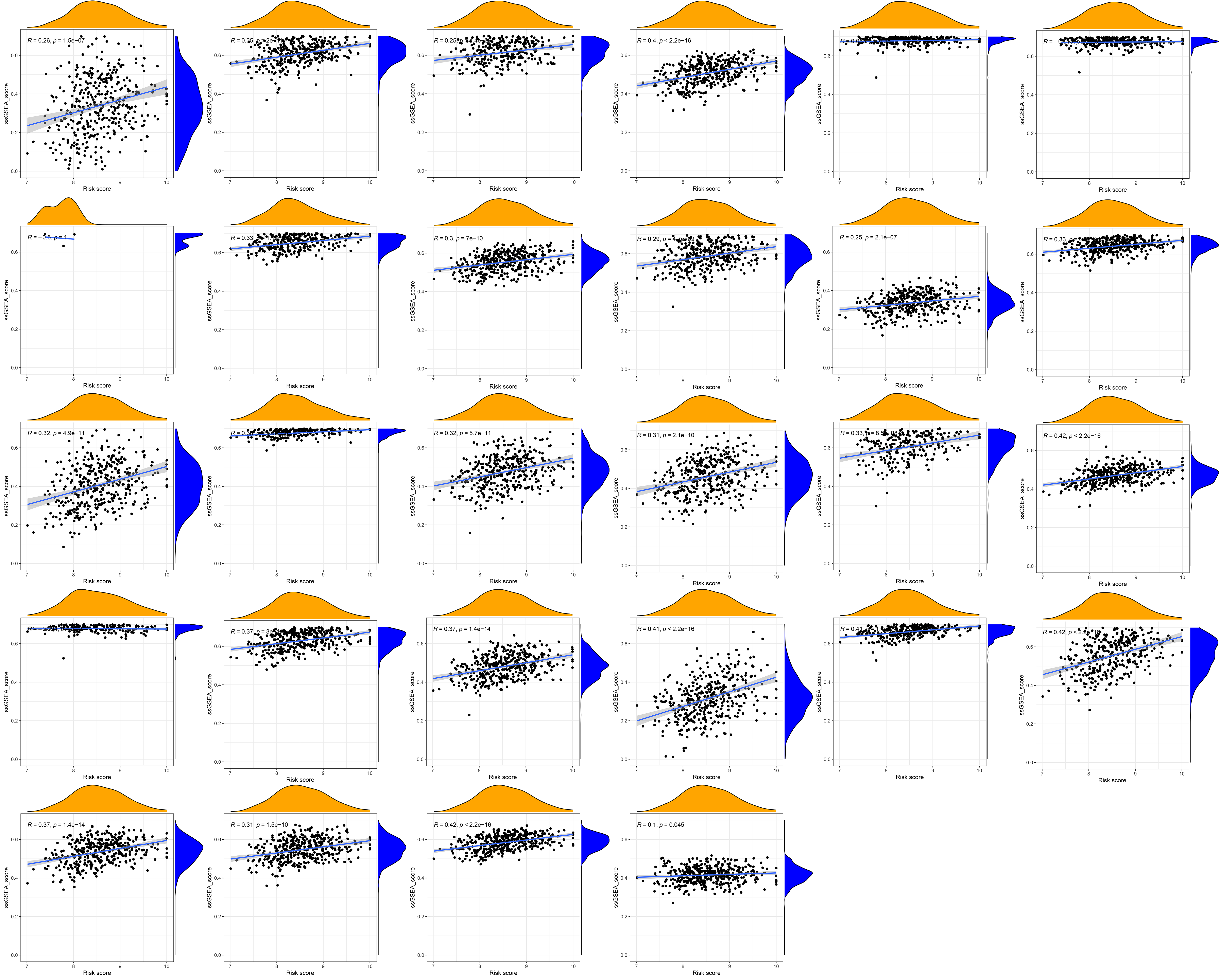

Supplement: Supplementary Figure 2 — Correlation analysis of signature with 28 immune cell infiltrates. [file Image_2.tif]

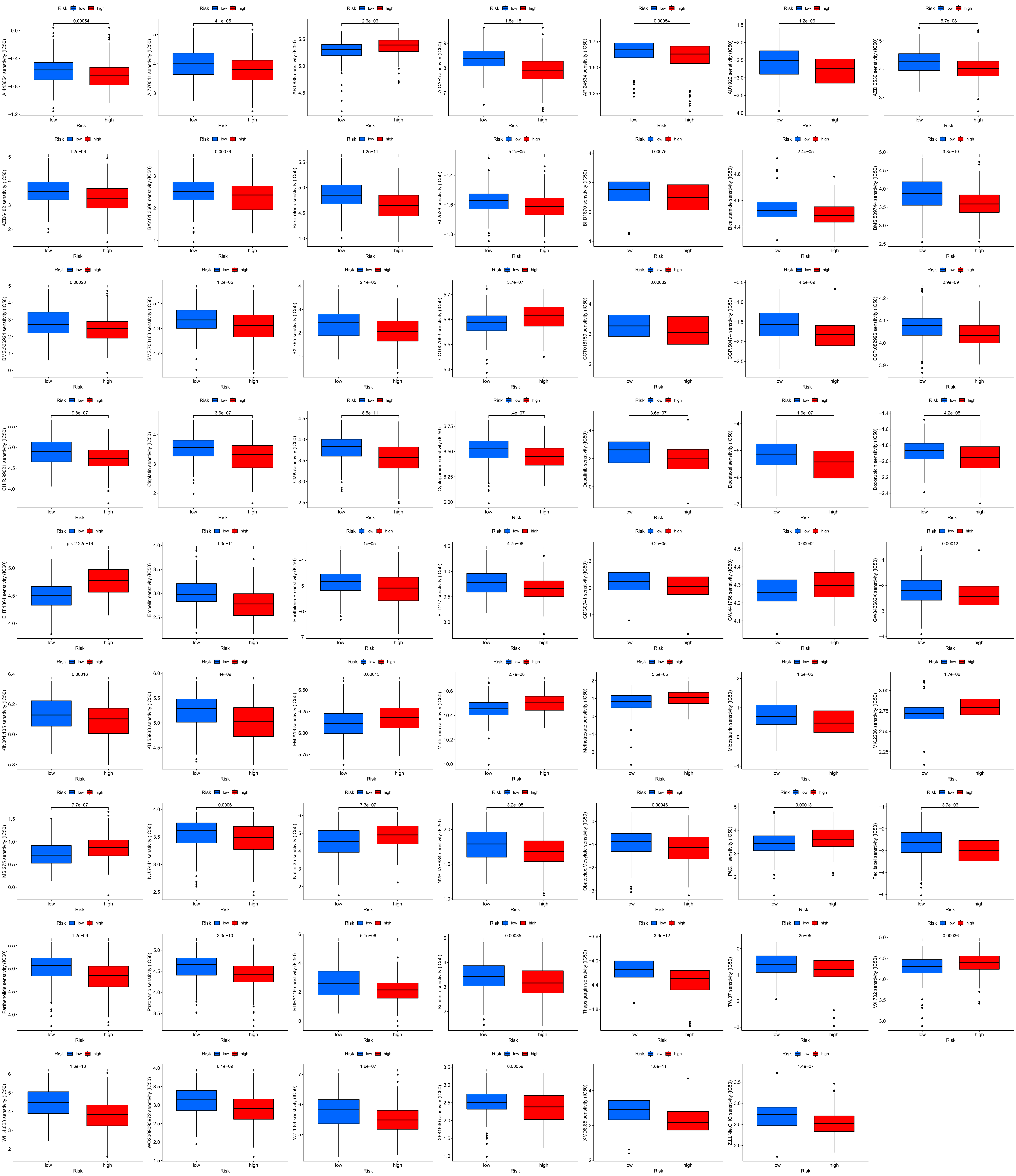

Supplement: Supplementary Figure 3 — Signature and drug sensitivity correlation analysis. [file Image_3.tif]
